# Effects of external hand force modeling on validity of inverse analysis of lifting

**DOI:** 10.1016/j.isci.2025.114146

**Published:** 2025-12-10

**Authors:** Eunsik Choi, Ilseung Park, Jeongin Moon, Jangwhan Ahn, Jooeun Ahn

**Affiliations:** 1 Department of Physical Education, Seoul National University, Seoul, Republic of Korea; 2 Department of Mechanical Engineering, Carnegie Mellon University, Pittsburgh, PA, USA; 3 Department of Mechanical and Industrial Engineering, University of Massachusetts Lowell, Lowell, MA, USA; 4 Department of Biomedical Engineering, University of North Carolina at Chapel Hill, Chapel Hill, NC, USA; 5 Soft Robotics Research Center, Seoul National University, Seoul, Republic of Korea; 6 Institute of Sport Science, Seoul National University, Seoul, Republic of Korea

**Keywords:** Biological sciences, Biomechanics

## Abstract

Manual lifting tasks pose the risk of musculoskeletal injury, including damage to lumbar intervertebral discs. To assess the risk, spinal joint reaction forces are often estimated using musculoskeletal simulations. However, such analyses require information of external hand force and moment (EHF&M), which has mostly been estimated using simplified models without sufficient validation. This study evaluates the validity of two common EHF&M models by comparing their analysis outcomes to those resulting from directly measured EHF&M. Simplified models yielded negligible errors during the middle phase of symmetric lifting and lowering but caused substantial errors at the beginning and end of each task, leading to inaccurate estimation of peak spinal joint reaction forces. These findings highlight the limitations of simplified models in evaluating mechanical factors of injury risk. The measurement platform and analysis method developed in this study can also contribute to validating EHF&M modeling for other tasks and improving assessment accuracy.

## Highlights

• Simplified EHF&M models are accurate during the middle phase of load handling
• Simplified approaches misidentify the timing and magnitude of peak joint reaction force
• These errors hinder effective design and control of lifting assistive devices
• Refined models that robustly detect grip-deposit transition are necessary

## Graphical abstract

**Figure:**
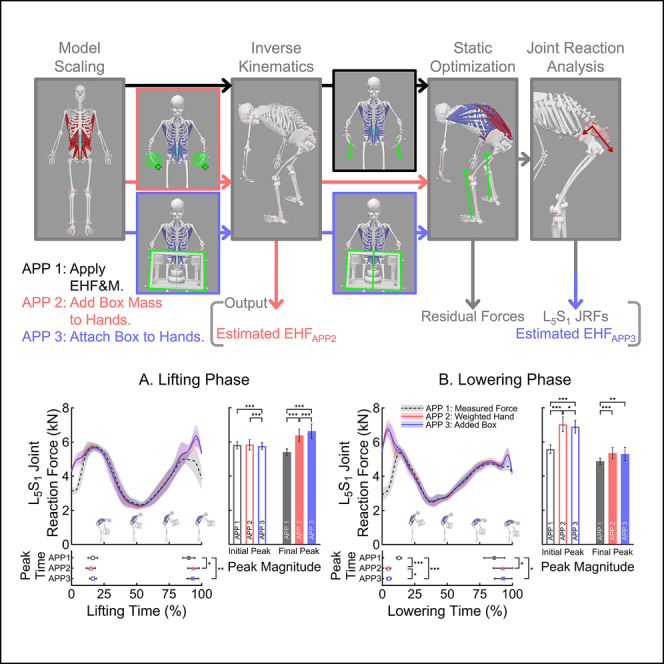

## Introduction

Workers in labor-intensive environments face an elevated risk of musculoskeletal injuries from repetitive manual lifting tasks of heavy loads.^1^^,^^2^^,^^3^^,^^4^ Epidemiologic studies have shown that workers in occupations involving frequent lifting of heavy loads encounter a significantly higher risk of acute lumbar intervertebral disc injuries compared to those in non-lifting occupations.^1^^,^^5^ The high joint reaction forces (JRFs) exerted on the lumbar spine during lifting tasks are transmitted to the intervertebral discs, potentially causing deformation or rupture and thereby increasing the risk of low back pain.^4^^,^^5^^,^^6^ To diagnose such risk and propose adequate intervention, it is essential to examine lumbar spinal joint reaction forces and moments (JRF&M), whose magnitudes are widely used as biomechanical indicators of injury risk in lifting tasks.^3^^,^^7^^,^^8^ However, direct *in vivo* measurement of JRFs is currently challenging without invasive methods,^9^ and therefore, the musculoskeletal simulation based on inverse analysis is universally adopted as an effective alternative for estimating JRFs.^10^^,^^11^

Multiple studies have put efforts to enable more accurate and efficient estimations of spinal JRF&M during lifting tasks. Sophisticated lumbar joint and muscle models have been developed to achieve more accurate and realistic estimation of spinal JRF&M,^12^^,^^13^^,^^14^ whereas simplified models of external hand force and moment (EHF&M) were also developed and adopted.^12^^,^^15^^,^^16^^,^^17^^,^^18^^,^^19^^,^^20^^,^^21^^,^^22^ Although simplifying EHF&M may limit the accuracy of the estimates of spinal JRF&M,^15^ the simplified models have been commonly selected by researchers due to the advantages in avoiding the additional installation of expensive force transducers and facilitating experiments in out-of-the-lab environments.^16^

The most widely selected approach to simplifying EHF&M is incorporating the mass of the handling object into the model. Several studies adopted this approach by constructing a box model with the information of its mass, volume, and inertial properties.^17^^,^^18^^,^^19^ This approach enables the analysis of dynamic interactions between the human and the box by creating joints between the two. Other studies adopted a further simplified approach by adding the mass of the handling box to the hands within the human model.^12^^,^^15^^,^^20^ As both mass-incorporated modeling approaches assume that the object remains in contact with the hands, their analysis is valid only during the handling phase. Therefore, a grip event detection process is necessary to identify the handling phase from the inverse analysis. Apart from these two commonly selected approaches, Faber et al. estimated external hand forces (EHFs) using measured ground reaction force and moment, human weight, and each body kinematics.^21^ Muller et al. also estimated EHFs by minimizing a cost function under the constraints of the equations of motion.^22^

Although various simplification approaches have been proposed, a systematic validation of such approaches in musculoskeletal simulations for lifting tasks has not been performed. In inverse analysis, the accuracy of the estimation of internal loads including spinal JRFs is directly affected by the accuracy of the information about external contact forces, which serve as input data.^23^^,^^24^ Therefore, thorough validation must precede any simplification of EHF&M. A few studies have compared estimated EHFs with measured data,^21^^,^^22^ but they either estimate spinal JRFs using musculoskeletal models without trunk muscles or did not estimate these forces at all. An attempt has been made to evaluate the validity of simplified EHF&M approaches for estimating spinal JRFs in musculoskeletal simulation,^16^ but validation was not possible due to the absence of measured EHF&M.

In this study, we aim to assess the validity of the widely used EHF&M modeling approaches. We devised a box whose handles are made of 6-axis load cells that directly measure EHF&M during lifting tasks. We estimated L_5_S_1_ JRFs, which are the highest forces among lumbar spine joints and therefore used as the most representative indicator of spinal safety.^23^ The estimated JRFs were compared across three conditions: directly using the measured EHF&M (APP 1), simplified approach by adding the mass to the hands (APP 2), and simplified approach by creating joints between hands and the box model (APP 3). To ensure the validity of our estimation, we additionally compared the measured and estimated EHFs and monitored the residual forces at the ground-pelvis joint.^24^^,^^25^^,^^26^

## Results

### RMSE of estimated EHFs and L_5_S_1_ JRFs

**Figure 1:**
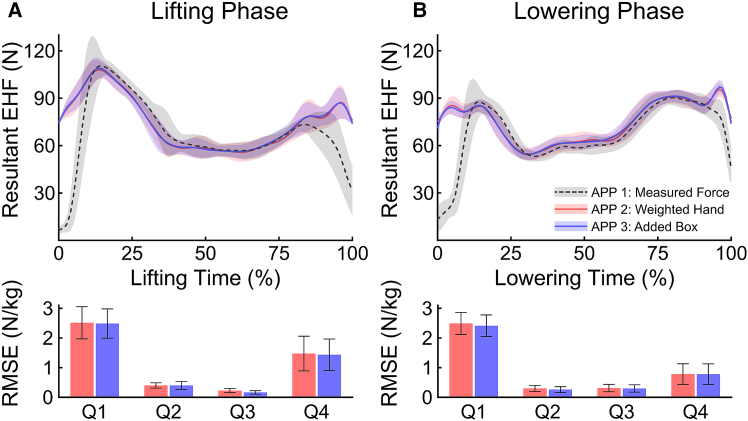
Measured and estimated EHFs with RMSEs during 20 lifting and lowering phases (A) corresponds to the lifting phase, and (B) corresponds to the lowering phase. The line plots show the mean measured force (APP 1, gray dashed) and estimated forces (APP 2, red; APP 3, blue), with shaded regions representing standard deviations. The bottom bar plots present the mean and standard deviation of RMSEs for APP 2 and APP 3 relative to APP 1 across quarter intervals (Q1–Q4), normalized by box mass.

Given the high bilateral symmetry of the EHFs (as shown in Table S1), the right-hand data were considered representative of both sides. Table 1 and Figure 1 show the difference between the measured and estimated EHFs of right hand during lifting and lowering phases. While APP 2 and APP 3 performed well during the mid-intervals (25%–75%), their accuracy declined at the beginning and final intervals of both phases. For the lifting phase, the root mean squared error (RMSE) of EHF in the 0%–25% interval was 37.8 N (2.51 N/kg) for APP 2 and 37.4 N (2.49 N/kg) for APP 3. Similarly, in the 75%–100% interval, the RMSEs of EHF for APP 2 and APP 3 were 22.2 N (1.48 N/kg) and 21.6 N (1.44 N/kg), respectively. In contrast, during the mid-intervals (25%–75%), RMSE of EHF remained below 6.0 N for both approaches. In the 25%–50% interval, RMSEs for APP 2 and APP 3 were both 6.0 N, corresponding to 0.40 N/kg. In the 50%–75% interval, the RMSEs for APP 2 and APP 3 were 3.4 N (0.23 N/kg) and 2.5 N (0.17 N/kg), respectively. The lowering phase exhibited similar trends. In the 0%–25% interval, the mean RMSE of EHF reached 37.4 N (2.49 N/kg) for APP 2 and 36.3 N (2.41 N/kg) for APP 3. In the 25%–50% interval, RMSEs for APP 2 and APP 3 were 4.5 N (0.30 N/kg) and 4.0 N (0.26 N/kg), respectively. In the 50%–75% interval, the RMSEs for APP 2 and APP 3 were 4.7 N (0.31 N/kg) and 4.5 N (0.30 N/kg), respectively. At the 75%–100% interval, the RMSEs were 11.8 N (0.78 N/kg) for both approaches.

**Table 1:** RMSE of estimated EHF during lifting and lowering phases, divided into four intervals (Q1: 0%–25%, Q2: 25%–50%, Q3: 50%–75%, and Q4: 75%–100%)

Dependent variable	Phase	EHF&M modeling approach	RMSE							
			Q1 (0%–25%)		Q2 (25%–50%)		Q3 (50%–75%)		Q4 (75%–100%)	
			N	N/kg	N	N/kg	N	N/kg	N	N/kg
Resultant EHF	Lifting	APP 2	37.8 (8.2)	2.51 (0.54)	6.0 (1.3)	0.40 (0.09)	3.4 (1.1)	0.23 (0.07)	22.2 (8.8)	1.48 (0.58)
		APP 3	37.4 (7.4)	2.49 (0.50)	6.0 (2.0)	0.40 (0.13)	2.5 (0.9)	0.17 (0.06)	21.6 (7.9)	1.44 (0.53)
	Lowering	APP 2	37.4 (5.6)	2.49 (0.37)	4.5 (1.5)	0.30 (0.10)	4.7 (1.9)	0.31 (0.12)	11.8 (5.2)	0.78 (0.35)
		APP 3	36.3 (5.5)	2.41 (0.37)	4.0 (1.5)	0.26 (0.10)	4.5 (1.9)	0.30 (0.12)	11.8 (5.2)	0.78 (0.35)
Resultant L_5_S_1_ JRF	Lifting	APP 2	891.1 (234.6)	11.86 (3.12)	208.5 (53.6)	2.77 (0.71)	96.9 (28.4)	1.29 (0.38)	881.3 (362.0)	11.73 (4.82)
		APP 3	884.2 (189.5)	11.76 (2.52)	170.8 (71.6)	2.27 (0.95)	79.3 (21.4)	1.06 (0.28)	943.7 (307.5)	12.56 (4.09)
	Lowering	APP 2	1,987.1 (360.6)	26.44 (4.80)	84.6 (25.1)	1.13 (0.33)	92.0 (28.2)	1.22 (0.38)	328.3 (157.1)	4.37 (2.09)
		APP 3	1,948.2 (347.1)	25.92 (4.62)	113.4 (42.0)	1.51 (0.56)	87.1 (23.7)	1.16 (0.32)	293.2 (154.4)	3.90 (2.05)

Results are presented in both absolute values (N) and normalized by box mass (N/kg) for APP 2 and APP 3. Data represented as mean and standard deviation of 20 trials. Standard deviations are shown in parentheses.

The estimated L_5_S_1_ JRFs showed a similar trend of RMSE to that of the estimated EHFs. For the lifting phase, the mean RMSE of L_5_S_1_ JRFs in the 0%–25% interval was 891.1 N (11.9 N/kg) for APP 2 and 884.2 N (11.8 N/kg) for APP 3. Similarly, in the 75%–100% interval, the RMSEs for APP 2 and APP 3 were 881.3 N (11.7 N/kg) and 943.7 N (12.6 N/kg), respectively. In contrast, in the 25%–50% interval, the RMSEs for APP 2 and APP 3 were 208.5 N (2.77 N/kg) and 170.8 N (2.27 N/kg), respectively. In the 50%–75% interval, the RMSEs for APP 2 and APP 3 were 96.89 N (1.29 N/kg) and 79.31 N (1.06 N/kg), respectively. For the lowering phase, the mean RMSE in the 0%–25% interval was 1,987.1 N (26.4 N/kg) for APP 2 and 1,948.2 N (25.9 N/kg) for APP 3. In the 25%–50% interval, the RMSEs for APP 2 and APP 3 were 84.56 N (1.13 N/kg) and 113.4 N (1.51 N/kg), respectively. In the 50%–75% interval, the RMSEs for APP 2 and APP 3 were 92.03 N (1.22 N/kg) and 87.10 N (1.15 N/kg), respectively. In the 75%–100% interval, the RMSEs for APP 2 and APP 3 were 328.3 N (4.37 N/kg) and 293.2 N (3.90 N/kg), respectively.

### Peaks of resultant L_5_S_1_ JRFs

**Figure 2:**
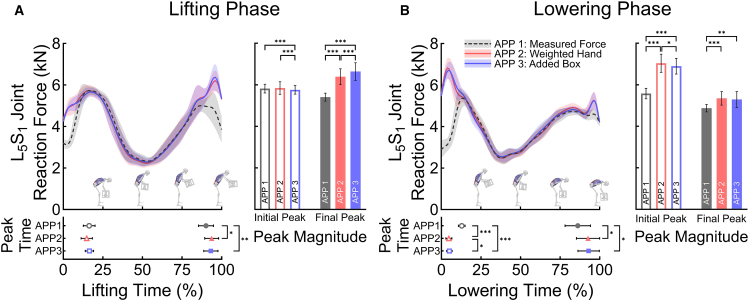
Profiles of estimated L5S1 JRFs, and their peak magnitude and time during 20 lifting and lowering phases (A) Corresponds to the lifting phase, and (B) corresponds to the lowering phase. The line plots show the mean and standard deviation of estimated L_5_S_1_ JRFs across three EHF&M modeling approaches (APP 1, gray dashed; APP 2, red; APP 3, blue). Peak times and magnitudes are identified for the initial and final peaks in each phase. The bottom dot plots display the mean and standard deviation of peak times, and the right bar plots display the mean and standard deviation of peak magnitudes across approaches, with significant post-hoc differences indicated (∗ p < 0.05, ∗∗ p < 0.01, ∗∗∗ p < 0.001).

Figure 2 illustrates the mean and standard deviation of resultant L_5_S_1_ JRFs with their peak times and magnitudes. For the initial stage of lifting phase, the mean and standard deviation of peak times were 16.40% ± 3.72% for APP 1, 14.85% ± 3.57% for APP 2, and 16.55% ± 2.65% for APP 3. The peak magnitudes were 5,814.70 ± 211.48 N for APP 1, 5,840.81 ± 304.33 N for APP 2, and 5,757.09 ± 207.76 N for APP 3. For the final stage of lifting phase, the peak times and magnitudes were 90.10% ± 4.70% and 5,404.73 ± 192.47 N for APP 1, 93.70% ± 4.40% and 6,386.11 ± 380.84 N for APP 2, and 93.15% ± 4.49% and 6,635.44 ± 424.04 N for APP 3. For the lowering phase, the peak times and magnitudes of the initial stage were 12.85% ± 2.08% and 5,575.55 ± 253.97 N for APP 1, 4.70% ± 1.89% and 7,035.37 ± 438.20 N for APP 2, and 5.05% ± 1.73% and 6,895.73 ± 377.51 N for APP 3. The peak times and magnitudes of the final stage were 86.15% ± 8.00% and 4,871.36 ± 178.59 N for APP 1, 92.65% ± 7.33% and 5,343.95 ± 323.10 N for APP 2, and 93.00% ± 6.74% and 5,291.78 ± 382.10 N for APP 3.

Figure 2 also presents the statistical results of peak time and magnitude for the resultant L_5_S_1_ JRFs of both the initial and final peaks during the lifting and lowering phases. Significant differences were noted across EHF&M modeling approaches in initial peak magnitudes of the lifting phase (F(1.45, 27.49) = 211, *p* < 0.001), final peak times and magnitudes of the lifting phase (F(1.55, 29.41) = 10.7, *p* < 0.001; F(1.17, 22.29) = 200, *p* < 0.001), and initial and final peak times and peak magnitudes of the lowering phase (F(1.32, 25.12) = 1045, *p* < 0.001; F(1.19, 22.53) = 189, *p* < 0.001; F(2, 38) = 9.14, *p* < 0.001; F(1.09, 20.77) = 21.9, *p* < 0.001). Only for the initial peak times of the lifting phase, statistical significance was not concluded (F(2, 38) = 2.51, *p* = 0. 094).

Bonferroni post hoc analysis revealed significant differences in peak time at the final lifting phase between APP 1 and APP 2 and between APP 1 and APP 3 (*p* = 0.007; *p* = 0.003), whereas no significant difference was observed between APP 2 and APP 3 (*p* = 0.403). In the peak magnitude at the initial lifting phase, significant differences were found between APP 1 and APP 3 (*p* < 0.001) as well as between APP 2 and APP 3 (*p* < 0.001), but no significant difference was observed between APP 1 and APP 2 (*p* = 0.541). For peak magnitude at the final lifting phase, significant differences were observed across all conditions (all *p* < 0.001).

For the peak time at the initial lowering phase, significant differences were observed across all conditions (*p* < 0.001; *p* < 0.001; *p* = 0.014). Also, at the final lowering phase, significant differences were observed between APP 1 and APP 2 and between APP 1 and APP 3 (*p* = 0.010; *p* = 0.008), but no significant difference was found between APP 2 and APP 3 (*p* = 0.812). For the peak magnitude at the initial lowering phase, significant differences were observed across all conditions (*p* < 0.001; *p* < 0.001; *p* = 0.004). At the final lowering phase, significant differences were found between APP 1 and APP 2 as well as between APP 1 and APP 3 (*p* < 0.001; *p* = 0.002), whereas no significant difference was observed between APP 2 and APP 3 (*p* = 0.220).

### Vertical residual forces

**Figure 3:**
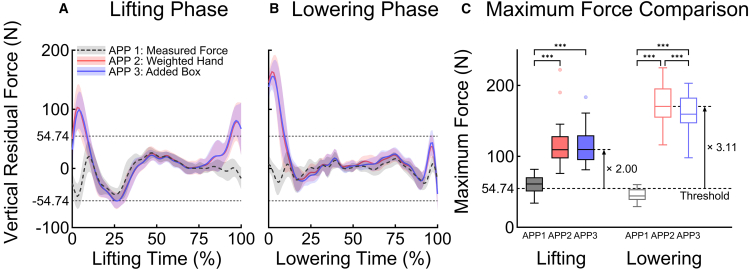
Optimized vertical residual forces and their maximum values during 20 lifting and lowering phases The line plots, (A) and (B), show the mean and standard deviation of optimized vertical residual forces across three EHF&M modeling approaches (APP 1, gray dashed; APP 2, red; APP 3, blue) during lifting and lowering, respectively. The horizontal dashed lines indicate the identified threshold. The boxplot, (C), displays maximum vertical residual forces across approaches, with significant post hoc differences indicated (∗∗∗*p* < 0.001). Boxes represent the interquartile range (25th–75th percentile), with whiskers extending to the most extreme data points within one-and-a-half times the interquartile range according to Tukey’s method. Red and blue dots represent outliers observed during the lifting phase in APP 2 and APP 3, respectively. Median maximum forces from APP 2 and APP 3 exceed the threshold by factors of approximately 2.00 and 3.11 during lifting and lowering phases, respectively.

Figure 3 illustrates the results of the vertical residual forces with their threshold derived from net external forces. The thresholds’ mean and standard deviation, calculated using ground reaction force and EHF, were 54.74 ± 1.10 N for the peak and 38.80 ± 0.33 N for the root mean square (RMS). The RMS values of all repetitions in APP 1 were below the RMS threshold. Although the averages of APP 2 and APP 3 exceeded the RMS threshold (43.02, 42.38 N in the lifting phase and 46.71, 46.03 N in the lowering phase), multiple repetitions complied with the thresholds. In contrast, neither APP 2 nor APP 3 stayed below the peak threshold at any repetition. Furthermore, the averages of APP 2 and APP 3 surpassed the peak threshold by more than twice their value.

Significant differences were observed across EHF&M modeling approaches in peak magnitude and RMS during the lifting phase (F(1.08, 20.56) = 46.9, *p* < 0.001; F(1.05, 19.92) = 76, *p* < 0.001) and lowering phase (F(1.16, 22.07) = 350, *p* < 0.001; F(1.02, 19.44) = 165, *p* < 0.001). Bonferroni post hoc analysis revealed significant differences between APP 1 and APP 2 as well as between APP 1 and APP 3 (all *p* < 0.001) (Figure 3). No significant difference was observed between APP 2 and APP 3 in peak magnitude and RMS during the lifting phase (*p* = 0.824; *p* = 0.575) and RMS during the lowering phase (*p* = 0.092), except for the peak magnitude during the lowering phase (*p* < 0.001).

## Discussion

**Figure 4:**
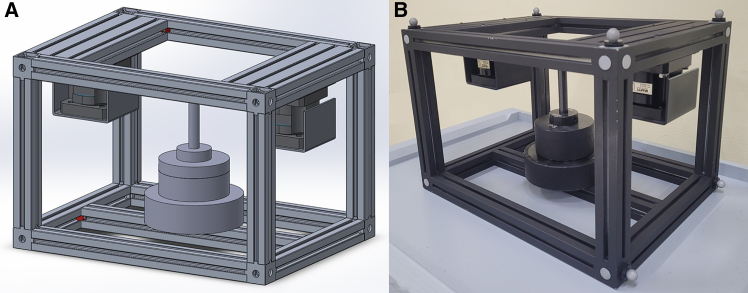
(A) A 3D CAD model and (B) a fabricated image of custom-built box with two load cells on handles

**Figure 5:**
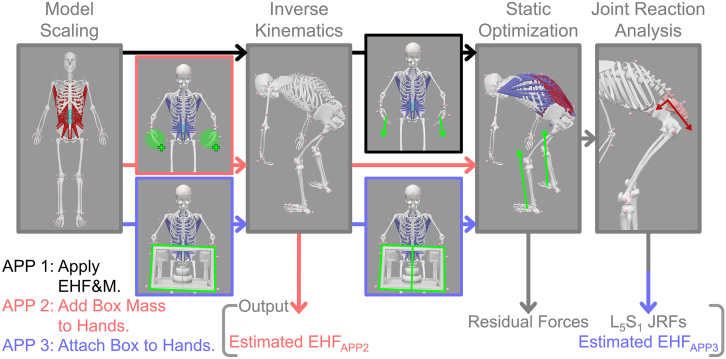
Block diagrams of simulation workflow and EHF&M modeling approaches The workflow follows four main steps: model scaling, inverse kinematics, static optimization, and joint reaction analysis. Three EHF&M modeling approaches are implemented. In APP 1, the measured EHF&M are directly applied (black arrows). In APP 2, half of the box mass is added to each hand (red arrows). In APP 3, two half-box models are attached to each hand, with the halves temporarily constrained as a single rigid body only during inverse kinematics (blue arrows).

**Figure 6:**
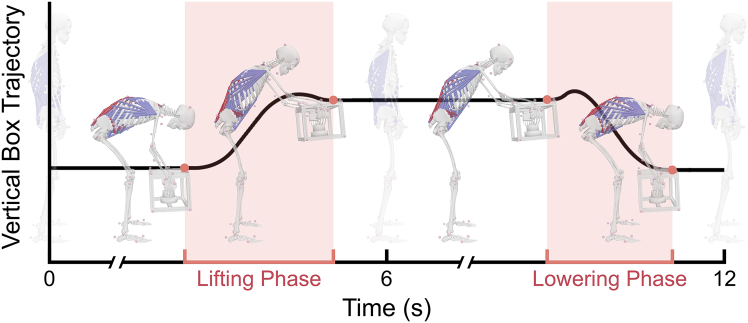
A representative profile of vertical box trajectory and corresponding lifting and lowering phases during a two-handed lifting task The black curvature shows the representative trajectory averaged across two top-anterior markers at the corners. The lifting and lowering phases (red shaded) are defined based on detected grip events (red dots).

In many previous studies, spinal JRFs have been estimated through musculoskeletal simulation. However, EHF&M, a critical input data in inverse analysis,^24^^,^^27^ have often been arbitrarily simplified without proper validation. In this study, we demonstrate how the most commonly used simple models of EHF&M impact the accuracy and dynamic consistency of musculoskeletal simulations. We adopted an integrated experimental and simulation framework, which is outlined in Figures 4, 5, 6, and Video S1, and explained in detail in the STAR Methods section.

Video S1. One cycle of two-handed symmetric lifting and lowering task

The accuracy of simplified EHF&M models varied substantially depending on the stage of the cycle of the lifting task. At the initial and final stages of the handling phase, simplified approaches produced significant errors in EHF estimates. These errors, normalized by the load’s mass, were considerably larger than the values reported in previous studies,^22^ which had already highlighted significant errors. At the same stages, their residual forces, which quantify the dynamic inconsistencies in the model,^24^^,^^25^^,^^26^ deviated significantly from the threshold. These findings suggest that the L_5_S_1_ JRF estimates at the beginning and end of the handling phase are not reliable.

Our results imply that the commonly used simplified EHF&M models can result in misleading interpretations. We show that L_5_S_1_ JRFs have peaks around the beginning and end of the handling phase, and this finding is consistent with the results of previous studies.^12^^,^^16^^,^^17^^,^^28^ The peak magnitude of the L_5_S_1_ JRF is a key indicator of the safety of a lifting task.^8^^,^^23^ Therefore, this indicator can be utilized for spinal injury prevention; it can be used to monitor whether the lifting motions comply with established guidelines?^8^ or to design the back support exoskeletons. However, we found that the peak magnitudes of L_5_S_1_ JRFs are prone to overestimation when the common simplified modeling approaches are employed (Figure 2). In addition, the simplified EHF&M models incorrectly identify the time point at which the peak of L_5_S_1_ JRF occurs (Figure 2). Similar patterns were observed across all lumbar joints, as shown in Figure S1.

Fortunately, since the peak spinal JRFs mostly tend to overestimate rather than underestimate (Table 1; Figure 2), it is hardly probable that the analysis based on the simplified models will conclude that unsafe lifting tasks are safe, at least for the symmetric handling tasks performed in this study. However, this overestimation remains detrimental to exoskeleton development. According to the safety guidelines used in recent studies employing musculoskeletal simulation,^3^^,^^7^^,^^29^ the lifting task is considered safe when the vertical L_5_S_1_ JRF remains below 3,400 N, whereas the task is considered unsafe when the JRF exceeds 6,400 N. Considering that the simplified EHF&M models overestimate the JRF values by more than 1,000 N (Figure 2), it is plausible that the estimation based on the simplified models imposes an excessive safety factor or over-specified design with unnecessary additional power, volume, or weight.

The misidentification of the timing of the peak JRF can also be detrimental. The control strategies of many recent assistive exoskeletons are based on bio-inspired profiles^30^^,^^31^^,^^32^^,^^33^ and therefore depend on precise detection of the timing of the peak loading or synchronization.^30^^,^^32^^,^^34^^,^^35^^,^^36^ In particular, parameters that determine the profile of the assistive torque are typically used as decision variables in optimizing the efficacy of an exoskeleton.^30^^,^^34^ Therefore, inaccurate prediction of the peak time of spinal JRF based on the simplified EHF&M models can lead to improperly tuned controllers and the decrease in the efficacy of the device.

Another important limitation of the common simplified EHF&M models is their inability to accurately identify the beginning and end of the handling phase. The beginning and end of handling need to be identified by detecting whether the load is applying forces to the hands.^16^^,^^22^^,^^37^ However, in the simplified models, the load’s mass is incorporated into the human model, and therefore the load’s gravitational force is always applied to the hands. Thus, the simplified models cannot encapsulate the gradual transitions of EHF&M values from or to zero. This limitation makes EHF estimates jerky at the boundaries of the handling phase and also degrades the accuracy of any analysis that depends on phase detection.

The inaccuracy of inverse analysis due to these mechanical limitations can inhibit the advancement of musculoskeletal modeling research aimed at predicting biomechanical dynamic mechanisms. Recent studies have demonstrated advances in musculoskeletal simulation, providing a more accurate representation of musculoskeletal modeling of internal loads during natural movement.^37^^,^^38^^,^^39^^,^^40^ In parallel, data-driven machine learning approaches have emerged for predicting injury risk of soft tissue (e.g., intervertebral disc, muscle, and ligament).^41^^,^^42^^,^^43^^,^^44^ If the challenges identified in our study can be overcome, the ongoing efforts toward more accurate musculoskeletal modeling will be strengthened and brought closer to practical implementation.

Our findings, however, reveal not only the limitations of the two common simplified EHF&M models but also their usefulness beyond convenience. In the middle phase of load handling, the simplified EHF&M models keep the estimated EHFs, L_5_S_1_ JRFs, and residual forces similar to those obtained from the direct measure of EHF&M. It is plausible that this accuracy in the middle of the handling phase results from the symmetric nature of the two-handed lifting that the participant performed in this study. Asymmetric load handling tasks, which involve, for example, rotation of the body in the transverse or frontal planes, may result in decreased accuracy even during the middle phase of handling. Nevertheless, the accuracy of the simplified EHF&M models during the middle phase of symmetric load handling suggests that the simplified approaches can be effective and efficient when estimating the spinal JRF during continuous and symmetric handling like holding or carrying a load with two hands for a prolonged period.

The common EHF&M models simplify contact forces, which are the critical inputs for an inverse analysis. In this study, we compare the estimation outcomes from such approach with those from direct measurement. This comparison was possible because we built a measurement system that can accurately collect EHF&M from the handle of the box. The devised system played an essential role in allowing us to perform inverse analyses of manual material handling tasks involving frequent grip events. This idea can be extended beyond occupational lifting to other motor tasks, including strength training exercises, which involve substantial joint and muscle loading and thus frequently induce injuries when performed with excessive load or improper posture.^45^^,^^46^^,^^47^^,^^48^ To assess the risk of such injuries and prevent their occurrence, it is necessary to predict the load on the joints and muscles according to the exercise load and posture. Embedding 6-axis load cells that directly measure EHF&M in the handling object or exercise equipment as handles will enable such prediction through an accurate and robust inverse analysis.

While our findings suggest that direct force measurement via embedded 6-axis load cells currently offers the most accurate solution for inverse analysis, such an approach is not always practical in real-world industrial settings. Therefore, our results highlight the need for developing advanced EHF&M modeling approaches that can approximate grip-deposit transitions and force magnitudes more effectively without relying on direct measurement.

### Limitations of the study

In this proof-of-concept investigation, we only recruited one young and healthy male participant and conducted the experiment and analysis. Recruiting a single participant was intended to isolate errors stemming purely from the modeling methods and minimize the confounding influence of inter-participant variability. Indeed, this approach with a single participant is consistent with that of multiple relevant previous studies, which successfully validated biomechanical modeling methods before applying them to larger populations.^16^^,^^49^ However, to make population-level inferences, further studies across a broader range of the population are necessary. This study also focuses exclusively on two-handed symmetric lifting and lowering tasks. Therefore, our findings may not be directly applicable to other asymmetric lifting and lowering tasks including torso twist. Future work needs to address these limitations by assessing the validity of EHF&M modeling for more diverse scenarios.

## Resource availability

### Lead contact

Requests for further information and resources should be directed to and will be fulfilled by the lead contact, Jooeun Ahn (ahnjooeun@snu.ac.kr).

### Materials availability

This study did not generate new materials.

### Data and code availability

• Raw and analyzed data have been deposited at GitHub: https://github.com/ces40320/BOX/tree/iScience-paper and are publicly available as of the date of publication. All other data reported in this paper will be shared by the lead contact upon request.
• All original code has been deposited at GitHub: https://github.com/ces40320/BOX/tree/iScience-paper and is publicly available as of the date of publication.
• Any additional information required to reanalyze the data reported in this paper is available from the lead contact upon request.

## Acknowledgments

This work was supported in part by the 10.13039/100020350Korea Health Technology R&D Project through the 10.13039/501100003710Korea Health Industry Development Institute (KHIDI) funded by the 10.13039/501100003625Ministry of Health & Welfare (no. HK23C0071), Industrial Strategic Technology (no. 20018157) and 10.13039/100020449Industrial Technology Innovation Program (no. 20007058, Development of safe and comfortable human augmentation hybrid robot suit) funded by the 10.13039/501100003052Ministry of Trade, Industry & Energy (MOTIE, Korea), and the 10.13039/501100003725National Research Foundation of Korea (NRF) grants funded by the 10.13039/501100014188Korean Government (MSIT) (no. RS-2023-00208052). The authors thank our laboratory members, Chihyeong Lee and Hyeonhee Jung, for their assistance with the installation of experimental facilities. We also extend our gratitude to Shinhye Park for administrative assistance in the procurement of equipment components.

## Author contributions

Conceptualization, E.C. and Jooeun Ahn; data curation, E.C.; formal analysis, E.C., I.P., and Jangwhan Ahn; funding acquisition, Jooeun Ahn; investigation, E.C., I.P., and Jangwhan Ahn; methodology, E.C., I.P., and Jangwhan Ahn; project administration, Jooeun Ahn; resources, E.C. and Jooeun Ahn; software, E.C. and I.P.; supervision, J.M. and Jooeun Ahn; validation, E.C., J.M., and Jooeun Ahn; visualization, E.C.; writing – original draft, E.C. and Jooeun Ahn; writing – review and editing, Jooeun Ahn.

## Declaration of interests

The authors declare no competing interests.

## STAR★Methods

### Key resources table

**Table:** 

REAGENT or RESOURCE	SOURCE	IDENTIFIER
**Deposited data**		

Human lifting data	The present study	GitHub: https://github.com/ces40320/BOX/tree/iScience-paper

**Software and algorithms**		

OptiTrack Motive 2.1.1	NaturalPoint Inc.	https://optitrack.com/software/motive
MATLAB R2021b	MathWorks Inc.	https://www.mathworks.com; RRID: SCR_001622
Lifting full-body model	Beaucage-Gauvreau et al.^12^	https://simtk.org/projects/lfbmodel
OpenSim 4.4	SimTK	https://simtk.org/frs/?group∖_id=91; RRID: SCR_002683
SolidWorks 2018	Dassault Systemes SolidWorks Corp.	https://www.solidworks.com; RRID: SCR_024908
Jamovi 2.3.28	The Jamovi project	https://www.jamovi.org; RRID: SCR_016142

### Experimental model and study participant details

One healthy young male (age: 30 years; weight: 75.16 kg; height: 174 cm) participated in the study to minimize interindividual variability. The participant had no history of neuromuscular, cardiovascular, and auditory disorders. This study was conducted in compliance with the ethical principles of the Declaration of Helsinki and after receiving approval from the institutional review board (IRB) of Seoul National University (IRB No. 2310/003–005). The participant provided informed written consent prior to participation, and he also signed informed written consent form for the publication of any identifying information or images in an online open-access publication.

#### Materials and equipment setup

In the experiment, a custom-built box with two handles was used as a load (Figure 4). The box, constructed from aluminum profiles for sturdy assembly, had two 6-axis load cells (MC3A6-1000, AMTI, Massachusetts, USA) installed on each handle. The box mass was set to 15 kg, which is below the recommended weight limit of industrial lifting and lowering guidelines.^50^ While the participant performed the instructed tasks, seventeen infrared cameras (OptiTrack Prime 13, NaturalPoint Inc., Oregon, USA) measured the three-dimensional position of the reflective markers affixed on the anatomical landmarks of the participant and eight corners of the box at a sampling frequency of 100 Hz. Also, two force plates (BMS400600, AMTI, Massachusetts, USA) and two 6-axis load cells measured GRF&M and EHF&M at a sampling frequency of 1000 Hz. The Motive software (OptiTrack Motive 2.1.1, NaturalPoint Inc., Oregon, USA) synchronized the measurements by controlling the equipment simultaneously.

### Method details

#### Experimental procedure

We provided the participant with athletic clothes to wear during the experiment. The participant moved the box from a shelf (height: 33 cm) in front of feet to a table (height: 78 cm) for lifting, and vice versa for lowering. Auditory triggers set at 10 bpm regulated the execution. One representative execution is shown in Video S1 of the supplemental information. The participant performed 20 repetitions of this two-handed lifting and lowering task.

#### Data pre-processing

All data measured in the experiments were unified in SI units. Spikes and gaps in the marker trajectories were removed using cubic spline interpolation. The expression of force, moment, and center of pressure data measured by the force transducers was matched to the global coordinate system (GCS) of the marker trajectory data. Since the 6-axis load cells were installed inside the box, these local coordinate systems (LCS) translated and rotated according to the box’s movements. In order to account for the movements of the LCS relative to the GCS, the translational trajectory and rotational angles of the box were calculated using eight markers attached to the box. The existing center of pressure data was adjusted using the box’s translational trajectory, and the existing force and moment data were transformed using the calculated rotational angles. The data signals were then processed using a 4th-order Butterworth low-pass filter with a cut-off frequency of 10 Hz. These processes were performed using MATLAB software (MATLAB R2021b, MathWorks Inc., MA, USA).

#### Musculoskeletal model

The lifting full-body (LFB) model is one of the few spine-related, open-access models available in the OpenSim database.^12^^,^^16^ Beaucage-Gauvreau et al. validated the LFB model’s kinematics and trunk muscle activations in two-handed stoop and squat tasks.^12^ The LFB model consists of 5 independent lumbar segments, allowing for the estimation of spinal JRF&M at 6 levels (from T_12_L_1_ to L_5_S_1_ joint).^12^ To accurately track hand kinematics while holding the box, the LFB model was modified by unlocking the degrees of freedom at the wrist joints, specifically in flexion-extension and deviation. Thus, the modified LFB model consists of 30 segments, 33 degrees of freedom, and 238 Hill-type muscle-tendon actuators. The upper and lower limbs, which do not include muscle-driven actuators, are driven by reserve actuators. Residual actuators at the free joint between the ground and pelvis are also included to account for dynamic inconsistency of the model.^24^^,^^25^^,^^26^

#### Simulation workflow

The modified generic LFB model was sequentially processed using four simulation tools available in OpenSim 4.4 (Figure 5).

#### Scaling

The generic model was scaled using the participant’s body mass and marker positions from a static posture. We used AddBiomechanics for this process.^51^

#### Inverse kinematics (IK)

Joint angles during the lifting and lowering motion were calculated by inputting marker trajectories into the scaled model.

#### Static optimization (SO)

Muscle activations and forces during the motion were estimated by solving the static optimization problem. The optimization minimizes the sum of squared muscle activations while satisfying the moment equilibrium at each joint. Given the joint angle data from inverse kinematics (IK) and the measured/estimated external forces and moments, the muscle forces were determined using Equations 1 and 2:

(Equation 1) $$\sum_{m=1}^{n}\left[a_{m}f\left(F_{m}^{0},l_{m},v_{m}\right)\right]r_{m,j}=\tau_{j}$$

(Equation 2) $$\text{minimize}\;J=\sum_{m=1}^{n}{\left(a_{m}\right)}^{2}$$

where, *n* is the number of muscles in the model, *a*_*m*_ is the activation level of muscle *m* at a discrete time step, $F_{m}^{0}$ is the maximum isometric force of muscle *m*, *l*_*m*_ is the length of muscle *m*, *v*_*m*_ is the shortening velocity of muscle *m*, $f\left(F_{m}^{0},l_{m},v_{m}\right)$ is the force-length-velocity surface, *r*_*m*,*j*_ is the moment arm of muscle *m* about the *j*^*th*^ joint axis, and *τ*_*j*_ is the joint torque acting about the *j*^*th*^ joint axis.

#### Joint reaction (JR) analysis

JRF&M were estimated based on the individual muscle forces determined through SO analysis.

#### EHF&M modeling approaches

##### Apply measured EHF&M in GCS (APP 1)

This approach used the pre-processed reaction forces, moments, and center of pressure data from 6-axis load cells as input for SO analysis. In APP 1, no additional process was required to estimate the EHF.

##### Add half-box mass to each hand (APP 2)

This approach simplifies the EHF&M by adding half the box mass to each hand body in the scaled LFB model. After this modeling process, we applied Newton’s law to estimate the EHF; the hand’s linear acceleration was substituted into Equation 3:

(Equation 3) $$EHF_{APP_{2}}=-\frac{m_{box}}{2}\left(a_{hand}-g\right)$$

where, *m*_*box*_, *a*_*hand*_, and *g* represent the mass of the box, linear acceleration of a hand, and gravitational acceleration, respectively. These linear accelerations were calculated by quadratic differentiation of the displacement data in the GCS obtained using OpenSim’s body kinematics tool.

##### Attach half-box bodies as weld joints (APP 3)

In this approach, a box model containing information of the mass, volume, center of mass, and mass moment of inertia was designed using 3D CAD software (SolidWorks 2018, Dassault Systèmes SolidWorks Corp., MA, USA) (Figure 4) and connected to both hands of the scaled LFB model via joints. Both joints were located on the third metacarpal head of each hand by calculating vertical distance from the origin of wrist joint’s LCS to the hand marker. The joint type was defined as a weld joint, which restricts translation and rotation in all directions. However, the OpenSim model fails when a child body is connected to more than one parent body.^52^ To avoid this error, we split the box model in half, connected each hand body with a weld joint, and connected the two-halves with a weld constraint, following the research of Akhavanfar et al.^16^ We removed the weld constraint from the model after the IK analysis because it rapidly increased the computation time of the SO analysis.^16^ Following this modeling process, the estimated EHFs in APP 3 were derived from the JRFs between the half-box and the hand using JR analysis.

#### Grip event detection

In APP 1, SO and JR analyses can be performed throughout the entire period because all external forces are known. In APP 2 and APP 3, by contrast, the hand and the box are integrated, and therefore, the accurate identification of the handling phases is restricted. We detected the grip events using MATLAB’s ‘findpeaks’ function on the average vertical trajectory of the two top-anterior markers at the corners. Based on these time points, the lifting and lowering phases were identified (Figure 6), and the simulations were performed for each handling phase.

#### Data processing

The handling phases of 20 two-handed symmetric lifting and lowering tasks performed by the participant were analyzed according to each modeling approach. In APP 1, one motion cycle was simulated, and the detected handling phases were extracted. In APP 2 and APP 3, the handling phases were split first, and the simulations were performed on these phases. All time-domain data were processed using cubic spline interpolation and normalized to 101 points (0–100%) of the data length for each handling phase.

#### RMSE of estimated EHFs and L_5_S_1_ JRFs

To directly compare the accuracy across different stages within a phase, we divided the phase into quarter intervals (0–25%, 25–50%, 50–75%, and 75–100%) and analyzed the root mean squared error (RMSE) of estimated EHFs. From the measured and estimated EHFs in the triaxial direction, we obtained the resultant EHFs by calculating the Euclidean norm. We calculated the RMSE between the two estimated EHFs and the measured EHF of the right hand, recorded using the 6-axis load cell. These RMSEs were then normalized by the mass of the box.

We additionally analyzed the RMSE of L_5_S_1_ JRFs. Similar to the EHF, the resultant forces were calculated from the triaxial L_5_S_1_ JRFs estimated by JR analysis in OpenSim. We quantified the RMSE between the JRF from APP 1 and JRFs from APP 2 and APP 3. These RMSEs were then normalized by the body mass of the participant.

#### Peaks of resultant L_5_S_1_ JRFs

For each handling phase, the participant performed two types of movements in sequence: shifting and placing the box. There are two major peaks of L_5_S_1_ JRFs per a handling phase, so we analyzed the time and magnitude of two peaks for each lifting and lowering phase.

#### Vertical residual forces

After SO analysis, we quantified the optimized residual forces at the ground-pelvis free joint. According to Hicks et al.,^24^ maintaining the peak and root-mean-square (RMS) of the vertical residual forces under 5% of the net external force is recommended to ensure dynamic consistency in simulation. We examined whether the vertical residual forces for each modeling approach satisfy these criteria.

### Quantification and statistical analysis

For the 20 lifting and lowering phases, we compared the dependent variables—mean peak time and peak magnitude of the resultant L_5_S_1_ JRFs, and each mean of peak and RMS of the vertical residual forces—across three approaches. One-way repeated measures analysis of variance (ANOVA) was used to assess significant differences in dependent variables. Bonferroni correction was selected for multiple pairwise comparisons. We tested sphericity of data using Mauchly’s test. When the assumption of sphericity was violated, the Greenhouse-Geisser correction was applied to adjust the degrees of freedom. The level of statistical significance was set at *p* < 0.05, and statistical analyses were performed using Jamovi 2.3.28.^53^
