# Supplementary material for: Effects of external hand force modeling on validity of inverse analysis of lifting
Source: iScience. 2025 Dec 10;29(1):114146. doi: 10.1016/j.isci.2025.114146 (PMC12800434; doi:10.1016/j.isci.2025.114146)
Supplement: Document S1. Figure S1 and Table S1 [file mmc1.pdf]

iScience, Volume *29*

Supplemental information

Effects of external hand force modeling on validity of inverse analysis of lifting

Eunsik Choi, Ilseung Park, Jeongin Moon, Jangwhan Ahn, and Jooeun Ahn

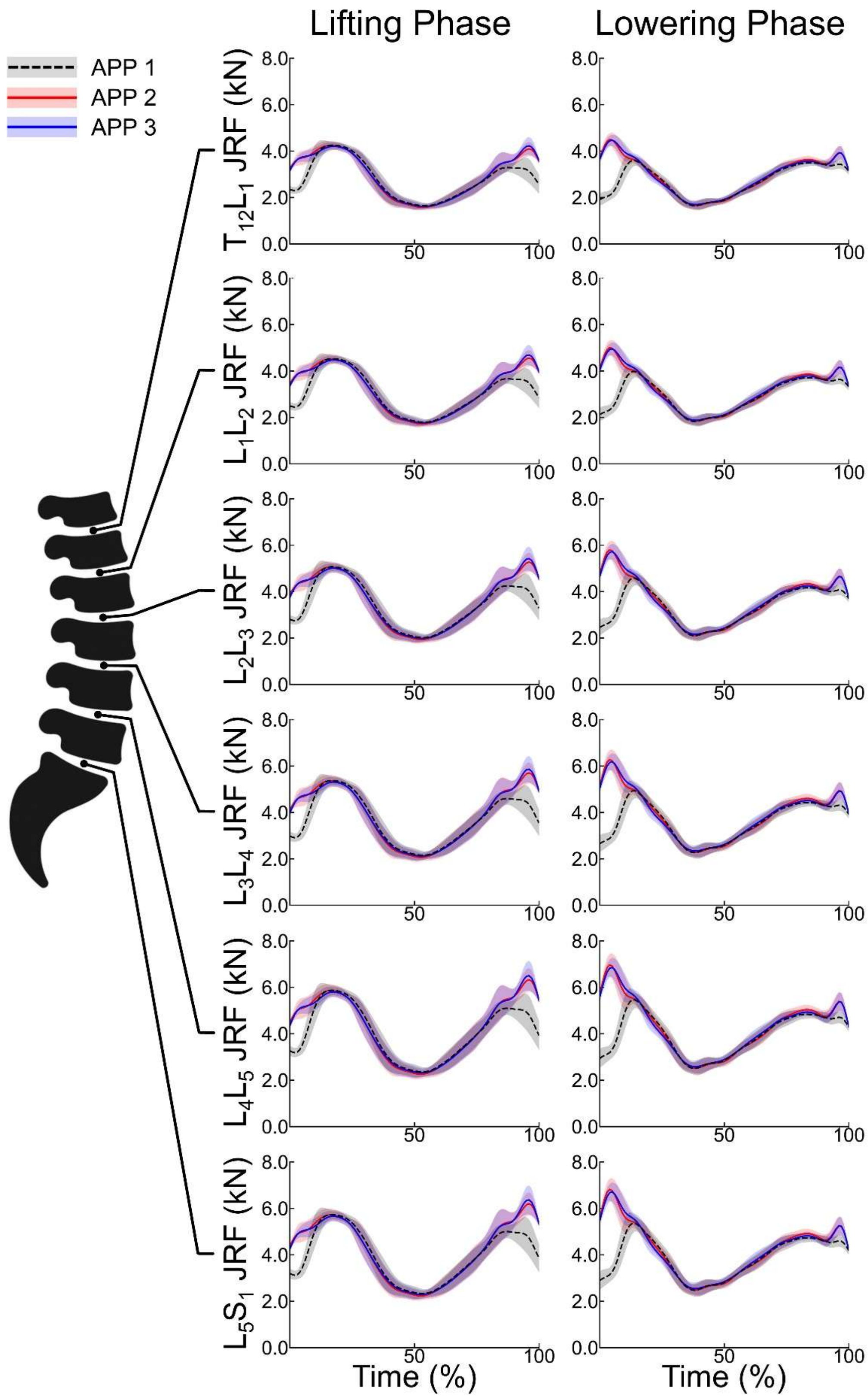

Figure S1. Profiles of estimated lumbar spinal JRFs during 20 lifting and lowering phases, related to Figure 2. The line plots show the mean and standard deviation of estimated joint reaction forces (JRFs) from $T_{12}L_1$ to L_5S_1 joints across three EHF&M modeling approaches (APP 1, gray dashed; APP 2, red; APP 3, blue).

Table S1. Mean, standard deviation, and statistical results of the differences in external hand forces between the left and right hands across each axis during 20 lifting and lowering tasks. We calculated the difference by subtracting the left hand's measurement from the right hand's. The statistical results of a paired t-test (n=20) are shown.

Condition		Mean Difference (N)	Std. Deviation (N)	Paired *t*	*p*
Lifting Phase	AP axis	-0.397	3.268	-0.779	0.374
	Vertical axis	-0.729	3.838	0.29	0.143
	ML axis	0.576	2.672	0.843	0.193
Lowering Phase	AP axis	-1.758	3.705	-1.605	0.142
	Vertical axis	0.674	4.088	1.619	0.22
	ML axis	2.065	3.176	2.894	0.142
